# Delineating mechanisms underlying parvalbumin neuron impairment in different neurological and neurodegenerative disorders: the emerging role of mitochondrial dysfunction

**DOI:** 10.1042/BST20230191

**Published:** 2024-04-02

**Authors:** Elizaveta A. Olkhova, Laura A. Smith, Bethany H. Dennis, Yi Shiau Ng, Fiona E. N. LeBeau, Gráinne S. Gorman

**Affiliations:** 1 Wellcome Centre for Mitochondrial Research, Faculty of Medical Sciences, Newcastle University, Framlington Place, Newcastle upon Tyne NE2 4HH, U.K.; 2 Translational and Clinical Research Institute, Faculty of Medical Sciences, Newcastle University, Framlington Place, Newcastle upon Tyne NE2 4HH, U.K.; 3 Biosciences Institute, Faculty of Medical Sciences, Newcastle University, Framlington Place, Newcastle upon Tyne NE2 4HH, U.K.; 4 NIHR Newcastle Biomedical Research Centre, Biomedical Research Building, Campus for Ageing and Vitality, Newcastle upon Tyne NE4 5PL, U.K.; 5 NHS Highly Specialised Service for Rare Mitochondrial Disorders, Newcastle upon Tyne Hospitals NHS Foundation Trust, Newcastle upon Tyne NE2 4HH, U.K.

**Keywords:** interneurons, mitochondrial dysfunction, molecular mechanisms, neurodegeneration, oscillations, parvalbumin

## Abstract

Given the current paucity of effective treatments in many neurological disorders, delineating pathophysiological mechanisms among the major psychiatric and neurodegenerative diseases may fuel the development of novel, potent treatments that target shared pathways. Recent evidence suggests that various pathological processes, including bioenergetic failure in mitochondria, can perturb the function of fast-spiking, parvalbumin-positive neurons (PV+). These inhibitory neurons critically influence local circuit regulation, the generation of neuronal network oscillations and complex brain functioning. Here, we survey PV+ cell vulnerability in the major neuropsychiatric, and neurodegenerative diseases and review associated cellular and molecular pathophysiological alterations purported to underlie disease aetiology.

## Introduction

Despite the majority of neurons consisting of excitatory glutamatergic cells, inhibitory neurons, which represent ∼20% of all cortical neurons, are indispensable for neuronal network regulation [1]. Discrete neuronal subpopulations that express the calcium-binding protein parvalbumin (PV) represent most of the gamma-aminobutyric acid (GABA) inhibitory neurons in the human and rodent brain [2,3]. PV+ expressing neurons possess unique biophysical properties including a fast-spiking firing rate with little accommodation or adaptation during depolarisation, which makes them crucial for inhibitory neuronal network regulation [4]. In the cerebral cortex, PV+ neurons are comprised of multiple types, the major ones constituting large basket cells and chandelier cells. These cells modulate the output of pyramidal neurons, by preferentially innervating their somata, or the axon initial segment (AIS), respectively, exerting powerful inhibition and controlling network synchrony [5,6].

Due to their fast-spiking properties, cortical PV+ neurons are critical for the generation of rhythmic fluctuation in the γ-band (∼30–100 Hz) frequencies [7], which in turn are crucial for sensory information processing and attention regulation [8–10].

Impairments in PV+ neuron function and excitatory/inhibitory (E/I) imbalance have been documented in a wide range of psychiatric and neurological disorders, including Alzheimer's disease (AD) [11], dementia with Lewy bodies (DLBs) [12], anxiety [13], schizophrenia [14], bipolar disorder [15], epilepsy [16], and more recently, primary mitochondrial disease (PMD) [17–19]. Whether the loss or dysfunction of PV+ neurons is contributing to the primary pathophysiological mechanisms, or rather is a consequence of these neurodegenerative disorders, remains to be fully elucidated.

This review aims to summarise current evidence from both human studies, and rodent *in vitro* and *in vivo* disease models, highlighting the role of PV+ neuronal dysfunction and associated pathophysiological mechanisms, including mitochondrial dysfunction, in several major neuropsychiatric and neurodegenerative diseases.

## Contribution of parvalbumin neuron dysfunction in epilepsy

Epilepsy is one of the most common neurological disorders worldwide and affects approximately 1 in 103 people in the U.K. (Epilepsy Research U.K.). It is characterised by recurrent, unprovoked seizure(s), caused by synchronous population discharges of excitatory neurons [20,21]. An imbalance of excitatory and inhibitory neuronal activity (E/I imbalance) is the fundamental mechanism underpinning neuronal hyperexcitability leading to seizures, highlighting the crucial role of the inhibitory neuronal population in regulating normal neuronal network activity to prevent seizures [22,23].

Despite a multitude of available anti-seizure medication (ASM), a third of patients’ seizures are not adequately controlled with treatment and these patients are at considerable risk of intractable epilepsy [24], defined as drug-resistant epilepsy (DRE). The majority of conventional ASMs mainly exert their mechanism of action by reducing neuronal firing, and not targeting the underlying cause of the epilepsy, which may explain the existence of a high rate of DRE. Therefore, delineating the pathological mechanisms implicated in epileptogenesis is crucial to inform future therapeutic strategies [25].

The most common form of adult focal epilepsy is temporal lobe epilepsy (TLE) which often involves the hippocampus and other limbic system structures [26]. TLE is typically associated with hippocampal sclerosis, which varies in severity and is characterised by specific patterns of neuronal loss, granular cell dispersion, gliosis, reorganisation of the neuronal architecture and alterations to interneurons [27]. In human post-mortem brain tissues obtained from patients with TLE and patients with cortical malformations that cause epilepsy, such as focal cortical dysplasia (FCD), the density of PV+ neurons is reported to be severely depleted [28,29]. Moreover, a selective loss of PV+ neurons in the subiculum [30] of patients with hippocampal sclerosis has been observed, despite an increase in the overall neuronal density in this brain region [28]. These studies suggest that the selective loss of PV+ neurons may be implicated in the pathogenesis of epilepsy by impairing inhibitory neurotransmission and promoting neuronal hyperexcitability. However, populations of other interneuron subtypes expressing calbindin (CB+), calretinin (CR+) and somatostatin (SST+) have also been reported to have an altered density and/or organisation in medial TLE [31–34], thus suggesting an involvement of multiple interneuron subtypes in the pathogenesis of epilepsy.

Within the experimental *in vivo* rodent models of epilepsy, a similar phenomenon of interneuron dysfunction has been described. A rat pilocarpine model of TLE exhibited a significant loss of PV+ neurons of more than 50% and a non-significant loss of SST+ neurons in the piriform cortex at 7 days, and 2 months post-status epilepticus [35]. Additionally, the same model demonstrated a rapid loss of PV+ neurons, which occurred during the acute and latent stages of epileptogenesis, before the initial spontaneous seizure onset, particularly in the dentate gyrus of the hippocampal formation [36]. This rapid PV+ cell loss was in contrast with the neurodegeneration of SST+ neurons, which occurred in the dentate gyrus later during the chronic stage of the epilepsy. These data suggest an early, preferential vulnerability of PV+ neurons to degeneration [36]. These findings were further corroborated by another study using the kainate-induced TLE mouse model, whereby PV+ neurons showed greater susceptibility to neurodegeneration in comparison to CR+ inhibitory neurons [37].

Interestingly, it was recently established that there is an interplay between the mammalian target of rapamycin (mTOR) pathway, which is involved in regulating metabolism, autophagy, mitochondrial structure and function, and PV+ neurons. A conditional knockout of the downstream repressor of the mTOR pathway specifically within PV+ neurons in mice resulted in an increased sensitivity to kainate- and pentylenetetrazole (PTZ)-induced epilepsy *in vivo* [38]. However, conditional knockout in excitatory neurons or GABAergic SST+ or vasoactive intestinal peptide-expressing (VIP+) interneurons did not lower the PTZ-induced seizure threshold [38], further supporting the roles of mTOR signalling in epilepsy and highlighting the individual cell type vulnerability of PV+ neurons.

Additional evidence implicating PV+ neurons in epilepsy stems from genetic disorders including Dravet syndrome, which is a rare genetic treatment-resistant epileptic encephalopathy, which begins in infancy or early childhood, frequently caused by the loss-of-function mutations in *SCN1A* [39]. This gene encodes the α-subunit of the voltage-gated sodium channel Na_v_1.1 which is predominantly expressed by PV+ neurons on their somata and axons [40,41]. Deletion of the *Scn1a* gene specifically from PV+ neurons *in vivo* results in spontaneous recurrent seizures in mice, which are not detected in transgenic mice with a *Scn1a* conditional knockout restricted to excitatory neurons [41]. It is proposed that the dysfunction of PV+ neurons due to Na_v_1.1 defects severely impairs inhibitory neurotransmission leading to neuronal hyperexcitability and seizures in Dravet syndrome [42].

The recent progress in the field of chemogenetic and optogenetic targeted activation or inhibition of certain neuronal subclasses has paved the way for greater understanding of epileptogenic mechanisms identified in living animals [43,44]. For instance, chemogenetic activation of PV+ neurons *in vivo* resulted in the attenuation of kainate-induced seizures by prolonging the latency to seizure onset and reducing the duration of the first generalised seizure, thereby decreasing the mortality rate of mice subjected to the intrahippocampal kainate administration [45]. Furthermore, *in vivo* optogenetic activation of hippocampal PV+ neurons, or PV+ Purkinje cells and molecular cell layer interneurons of the cerebellum, diminished seizure duration in mice [46,47]. Moreover, stimulation of PV+ neurons in the midline of the cerebellum additionally reduced the frequency of seizures which was not seen with optogenetic activation of PV+ neurons in the hippocampal formation [47]. Collectively, these studies highlight PV+ neurons as a promising therapeutic target in epilepsy.

## Inhibitory parvalbumin neuron involvement in primary mitochondrial disease

PMDs comprise the most common group of inherited metabolic disorders, characterised by extreme genotypic and phenotypic heterogeneity. PMDs can affect people at any age and can be caused by pathogenic variants in either nuclear DNA or mitochondrial DNA (mtDNA) [48]. There are genetic peculiarities of mtDNA variants, including multiple copies of mtDNA per nucleated cell, heteroplasmy (i.e. mixed mutated and wild-type mtDNAs in the tissues), threshold effect and genetic bottleneck [49]. Neurological manifestations are prevalent in PMDs, and may include cerebellar ataxia [50], extra-pyramidal movement disorders, progressive cognitive impairment [51], stroke-like episodes [52] and epileptic seizures [53], which are reported to affect up to 60% of paediatric and ∼23% of adult patients [54,55]. Status epilepticus in PMDs is often refractory or super-refractory to ASMs, including general anaesthetic agents, and intriguingly often demonstrate an occipital lobe predilection [52,56–58]. Stroke-like episodes in PMDs are subacute-onset evolving encephalopathic episodes associated with neurological and/or psychiatric symptoms, which are hypothesised to be driven by focal seizure activity [52,59,60]. Although the mechanisms underpinning epilepsy in PMDs have not been fully elucidated, there is mounting evidence demonstrating dysfunction of inhibitory interneurons and glial cells is implicated in neuronal hyperexcitability and seizure generation [17,18,61,62].

Previous neuropathological studies assessing post-mortem brain tissues from patients with PMD have demonstrated a severe loss of cortical GABAergic inhibitory interneurons, accompanied by extensive deficiencies in oxidative phosphorylation (OXPHOS) proteins in those remaining neurons [17]. Interestingly, comparison of the levels of OXPHOS protein deficiencies between neuronal subtypes revealed a more pronounced deficiency in inhibitory neurons compared with glutamatergic excitatory neurons in Alpers’ syndrome, a rare paediatric mitochondrial disease [63], thus suggesting a specific vulnerability of inhibitory neurons to metabolic impairment in PMD. Furthermore, a more recent study delineating the vulnerabilities of specific inhibitory neuron subtypes in Alpers’ syndrome revealed a preponderance of PV+ cortical neurons to degeneration [18]. Extensive OXPHOS protein deficiencies, indicative of mitochondrial dysfunction, were observed within remaining PV+ neurons and were more severe in comparison to CR+ neurons [18]. These findings suggest not only the preferential susceptibility of inhibitory neurons to dysfunction and degeneration in PMD, but also support the idea that PV+ neurons are particularly vulnerable to metabolic impairment, and thus likely have an important role in mitochondrial epilepsy.

Further evidence provided from acute hippocampal slices derived from rodents demonstrated that the application of OXPHOS complex I and complex IV inhibitors *in vitro* resulted in a marked reduction in PV+ fast-spiking neuronal firing and collapse of γ (30–80 Hz) frequency oscillations [64]. Since PV+ activity underpins γ frequency rhythms [65], this study provides further evidence of PV+ neuron vulnerability to mitochondrial dysfunction. However, mitochondrial impairment within astrocytes, induced through the application of an aconitase inhibitor, in combination with complex I and complex IV inhibition, has been shown to be required to induce interictal and ictal activities *in vitro* in rodent and human acute hippocampal slices, eliciting severe astrogliosis and loss of PV+ neurons as a result [61]*.* Thus, these studies suggest that PV+ neuron dysfunction, in conjunction with astrocytic impairments, drives mitochondrial seizure-like activity, at least in this model system.

**Figure 1.**
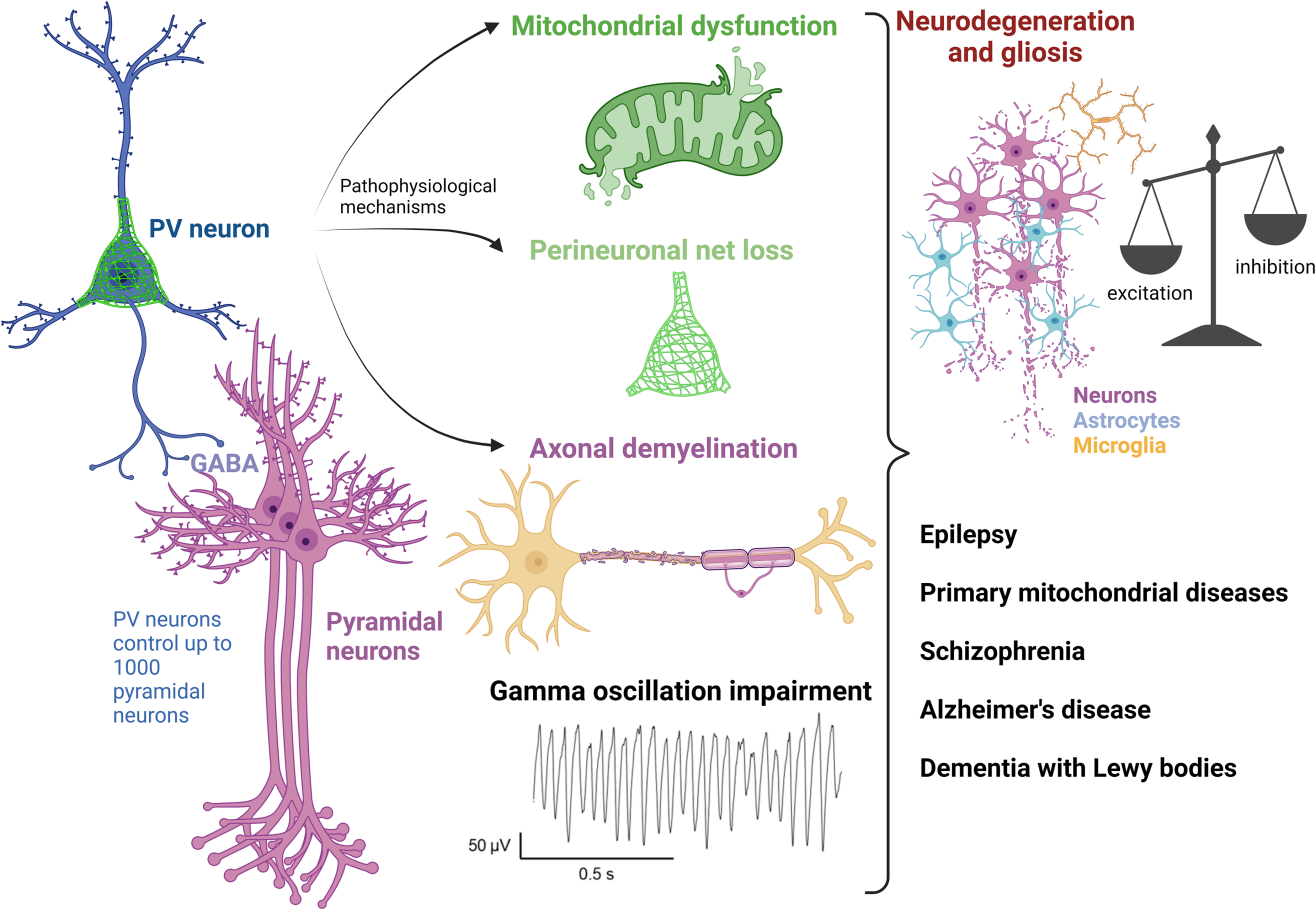
Dysfunction of PV+ neurons in neurological disorders. Schematic representation of the role of PV+ neurons and congruent pathophysiological mechanisms contributing to neurodegeneration and gliosis in the major psychiatric and neurodegenerative diseases, under review (created with biorender.com).

Multiple published *in vivo* models also provide further evidence implicating a vulnerability of PV+ neurons to mitochondrial dysfunction (Figure 1). An *in vivo* mouse model harbouring a mitochondrial complex IV subunit knockout specifically within PV+ neurons demonstrated altered electrophysiological properties of PV+ firing [66]. PV+ neurons harbouring mitochondrial impairment showed an increased adaptation (defined as progressive slowing of action potential discharge frequency in response to sustained excitation), and *in vivo* electrophysiological recordings demonstrated an increased power of γ frequency oscillations [66]. These mice also presented with a schizophrenia-like phenotype including impaired sociability and sensory information gating, although the authors did not report seizures in the transgenic animals [66]. Psychiatric comorbidities are prevalent in epilepsy [67], or vice versa, patients with schizophrenia are at a 4- to 5-fold higher risk of developing epilepsy than the general population [68]. PV+ neurons may be central to epilepsy and psychiatric comorbidities [69].

PV+ neuronal impairments may also underlie the pathogenesis of cerebellar ataxia in patients with PMDs. Purkinje neurons which, although GABAergic, are the sole output cells of the cerebellum, also express PV. Multiple post-mortem neuropathological studies have reported a severe depletion of Purkinje cells in the cerebellum from patients harbouring pathogenic variants in mtDNA and the nuclear DNA-encoded gene *POLG* [63,70,71]. Variable levels of mitochondrial OXPHOS protein deficiencies have also been reported in the remaining patient Purkinje cells [63,71]. The severe degeneration and dysfunction of Purkinje cells are hypothesised to alter the cerebellar circuitry, leading to neuronal hyperexcitability which may contribute to ataxic symptoms in patients with mitochondrial disease.

Combined, these studies support the notion that inhibitory cells are particularly vulnerable to mitochondrial dysfunction and together, may play an important role in the pathogenesis of debilitating neurological impairments observed in PMDs.

## Role of parvalbumin neurons in neuropsychiatric disorders

Schizophrenia is a neuropsychiatric disorder affecting around 24 million people worldwide (World Health Organisation, 2022). As a complex disorder, the cause of schizophrenia is a combination of genetic and environmental factors [72]. Clinically, schizophrenia presents with a range of symptoms, classified as either positive (delusions, hallucinations), negative (emotional and social dysfunction), or cognitive (impaired working memory and executive function) [73]. The positive symptoms of schizophrenia can be managed by antipsychotic medication, however, currently, there is no specific or targeted treatment for the negative and cognitive symptoms [74]. The mechanisms underlying these symptoms are still unclear and contribute significantly to the long-term burden of the disorder [75]. Cognitive symptoms develop during early adolescence and progress in severity into adulthood [76–78]. The glutamate hypothesis of schizophrenia suggests that cognitive impairment may be due to *N*-methyl-d-aspartate (NMDA) receptor (NMDA-R) hypofunction leading to disturbances in glutamate-mediated neurotransmission, especially in the prefrontal cortex (PFC) and hippocampus [79]. A key biomarker of these cognitive changes is high-frequency β (∼20–30 Hz) and γ (30–80 Hz) oscillations, rhythms which are generated by inhibitory, fast-spiking PV+ interneurons [80–82]. A favoured hypothesis suggests that loss of PV+, or reduced PV+ neuron function, causes disinhibition in excitatory–inhibitory neuronal circuits, leading to an E/I imbalance that may underlie the cognitive deficits of schizophrenia [83].

There is no single genetic cause for schizophrenia, but instead likely arises from polygenic mechanisms interacting with a variety of non-genetic factors [84]. Interestingly, two susceptible genes linked to schizophrenia include *NRG1*, the gene encoding neuregulin-1 (NRG1) [85], and *ERBB4*, which encodes receptor tyrosine kinase ERB-B4 [86–88]. ERB-B4 is a post-synaptic target of NRG1, primarily expressed in PV+ interneurons. Both NRG1 and ERB-B4 signalling are reportedly increased in the brains of schizophrenia patients [89]. Changes in ERB-B4, therefore, may underlie the pathological changes seen in GABAergic transmission and NMDA-R signalling seen in schizophrenia. A mutant mouse model, where *ErbB4* was specifically knocked out in PV+ interneurons (*PV-Cre;ErbB4^−/−^*), demonstrated a schizophrenia-like phenotype [90]. This included impairment in pre-pulse inhibition and working memory, as well as hyperactivity, highlighting the critical role of NRG1 in normal brain function [90]. Although PV+ neurons are the most widely studied neuronal subpopulation in schizophrenia, it is still not fully understood how PV+ cells are altered in the disorder.

In human post-mortem studies, differences in PV+ expression levels in neurons have been reported in multiple brain regions of schizophrenia patients. Although results vary, most studies suggest that patients with schizophrenia present with lower PV+ protein expression and *PVALB* mRNA in comparison with controls, in brain regions including the PFC [91–94], hippocampus [95] and entorhinal cortex [96]. Conversely, some studies found no significant change in PV+ expression [97–99], whilst PV+ expression was found to be elevated in the anterior cingulate cortex (ACC) of patients with schizophrenia [100]. Despite these variable findings, a recent transcriptomics study found down-regulation of genes involved in the OXPHOS system in PV+ neurons, suggestive of mitochondrial impairment in post-mortem ACC tissues of patients with schizophrenia [101]. This provides further links between PV+ impairment, mitochondrial dysfunction and schizophrenia.

In addition to changes in PV+ expression, aberrant β/γ frequency oscillations have also been demonstrated in patients with schizophrenia (Figure 1). During working-memory processing tasks, patients with schizophrenia showed reduced β/γ frequency activity during the memory retrieval phase of tasks [102] in comparison to healthy controls. Furthermore, where healthy controls demonstrated an increase in γ activity in response to tasks that required increased executive control and working memory load, patients with schizophrenia failed to demonstrate a similar increase [103,104].

In rodent models of schizophrenia, a loss of PV expression or PV+ cell dysfunction is also reported in both developmental and pharmacological models. Using the methylazoxymethanol acetate (MAM) neurodevelopmental model, PV+ density was reduced in the rat dentate gyrus [105], medial prefrontal cortex (mPFC), ACC and ventral subiculum [106]. Pharmacological treatment of rodents with NMDA-R antagonists, to mimic NMDA-R hypofunction, is another common experimental model. In healthy human subjects, NMDA-R antagonists, such as phencyclidine (PCP) and ketamine, were found to induce a full range of schizophrenia symptoms [107,108], and exacerbate cardinal symptoms in patients with schizophrenia, such as psychosis, hallucinations and cognitive impairment [109,110]. Furthermore, PCP treatment reduced PV+ expression in the prelimbic cortex when administered sub-chronically [111], as well as the cingulate cortex and hippocampus, when administered acutely [112]. Treatment of rodents with PCP also altered animal behaviour, inducing a schizophrenia-like phenotype including cognitive deficits such as in working memory [112], recapitulating cognitive symptoms in schizophrenia [113]. Knockdown of PV in rats produced negative schizophrenia symptoms such as social withdrawal and cognitive flexibility deficits [114], suggesting that PV itself plays a role in maintaining neuronal network homeostasis [115,116].

Recently, it has been proposed that redox dysregulation, NMDA-R hypofunction, neuroinflammation and mitochondrial bioenergetics deficits may result in vicious cycle of oxidative stress during brain development and have been implicated in the pathophysiology of schizophrenia [117]. Evidence for redox dysregulation is derived from transgenic animal models such as glutamate cysteine ligase modulatory subunit (*Gclm*) knockout. This model displayed evidence of glutathione deficit and exhibited striking oxidative stress, as exemplified by oxidative stress marker 8-oxo-2′-deoxyguanosine accumulation [118]. The oxidative stress resulted in a decrease in complex IV subunit COX6A2 expression in surviving PV+ neurons, suggesting diminished complex IV function and PV+ loss in the ACC [118]. These changes were accompanied by a reduction in mitophagy markers and an increase in miR-137, a noncoding microRNA which negatively regulates mitophagy [118]. Authors were able to reverse translate their findings to stratify patients with early psychosis by measuring blood exosome levels of miR-137 and COX6A2 as a proxy marker for PV+ integrity and mitochondrial function [118]. Patients with high levels of miR-137 and COX6A2 had worse cognitive task performance and reduced 40 Hz evoked power in response to auditory stimulus [118].

Overall data in both human studies and rodent models of schizophrenia demonstrate a link between PV+ cell dysfunction, mitochondrial impairment, the reduced generation of normal fast network oscillations and decreased cognitive function.

## Parvalbumin neuron deficits in Alzheimer's disease and dementia with Lewy bodies

AD is the most common form of dementia affecting ∼5% of the European population with prevalence increasing with age [119]. The main pathological features of AD are extracellular amyloid plaques and intracellular neurofibrillary tau tangles [11]. Lewy body dementia, which includes DLB and Parkinson's disease dementia, is caused by the abnormal aggregation of the synaptic protein α-synuclein and is the second most common form of dementia [120]. Both AD and DLB are progressive degenerative brain diseases which lead to synaptic dysfunction, network oscillation abnormalities and ultimately neuronal death [121–123].

The role of PV+ neurons in AD and DLB is important to understand because E/I imbalances occur leading to an increased risk of epilepsy in patients with AD [124]. Although most patients with sporadic AD do not present with overt clinical seizures, other indicators of abnormal network hyperexcitability, such as interictal discharges occur [125]. Moreover, seizures can be nocturnal or non-convulsive [126] and thus may be under-recognised and therefore under-reported. Sleep disturbances and epilepsy are known to be interlinked in AD and may exacerbate one another, having implications for memory deficits [127]. Cortical hyperexcitability is evident in patients with DLB who also have an increased risk of seizures or myoclonus [124], and often seizures may be subclinical [128]. In addition, patients with DLB frequently exhibit visual hallucinations and cognitive fluctuations [120], symptoms that may reflect changes in cortical network excitability [129]. Critically, AD patients with a history of hyperexcitability, clinically progress more rapidly [130,131], and patients with epilepsy are more likely to develop AD, suggesting a close association between abnormal excitation and dementia pathology.

Data from human post-mortem studies on the expression of PV+ cells in AD is, however, contradictory with reductions in PV reported [132], while others found no changes [133]. One study reported an association between the loss of PV+ neurons in the entorhinal cortex with neuropathological amyloid-β and tau burden [134]. Loss of PV expression has been reported in DLB post-mortem hippocampal tissue [12], and in primary visual cortical areas [135], which is similar to our observations in PMDs [18,19], while others found no changes [136]. However, many factors including the brain region studied, sex and disease stage could all contribute to the differences reported.

Evidence for changes in PV+ cells due to amyloid-β, tau or α-synuclein pathology is supported by studies using transgenic mouse models of AD and DLB. Multiple different murine models of AD exhibit cortical hyperexcitability in the form of seizures or interictal discharges, associated with impaired E/I balance [137,138]. Abnormal cortical excitability has also been reported in different transgenic mouse lines expressing either mutant or wild-type human α-synuclein both *in vivo* [139,140] and *in vitro* [141].

As with the human studies, details of changes in PV expression are variable in the murine models of AD, with some studies reporting reductions in PV immunoreactivity [142,143], while others found no change [144]. However, even within the same AD mouse model, regional differences in the impact of disease-related pathology on PV expression have been reported [142]. Data on PV expression in α-synuclein transgenic mice also varies with regional reductions [145,146], and with no change reported in young animals [141], which may not be surprising as at this age transgenic mice have not yet developed cognitive deficits.

In AD murine models PV+ neuronal activity has been reported as both increased [147] or decreased during specific oscillatory states [144,148]. Verret et al. found no change in PV expression levels, but reduced expression of the sodium channel Na_v_1.1 on the PV+ cells, leading to a reduced PV+ neuron firing rate, impaired γ oscillations, seizures and cognitive dysfunction. Using a different AD mouse model, Hijazi et al. [147] demonstrated that changes in PV cell activity were more complex, revealing a biphasic profile with increased activity at early disease stages, but reductions with more advanced disease. Further evidence to support the key role of PV+ neurons comes from studies in which specific restoration or modulation of PV+ neuron function was found to stabilise network excitation and restore oscillations and cognitive performance [149–151], although one recent study has challenged the proposed mechanisms underlying this effect [152].

PV+ neuron excitability is also regulated by extracellular matrix structures called perineuronal nets (PNNs) that surround the cell soma and proximal dendrites [153]. The PNN is one of the master regulators of E/I balance [154], and reduced PNN expression has been observed in human AD brains and rodent models [155,156], while astrocytes and resident macrophages of the brain called microglia also regulate PV+ cell excitability (Figure 1) [157,158]. In addition, PV+ cell axons are highly myelinated [159] and one recent report found evidence of demyelination of PV+ axons, but not excitatory neurons, at early disease stages in an AD mouse model [160]. While the causes of PV cell vulnerability in diseases have often focused on their high energy demands, as discussed in this review, changes in the PNN and/or myelination levels would also have profound effects on PV+ neuron's firing properties and function [161]. Moreover, demyelination, for instance in multiple sclerosis, can also preferentially affect inhibitory synapses and neurons, with the selective vulnerability of PV+ and SST+ cells [162].

As the deposition of amyloid-β and α-synuclein aggregation is activity-dependent, increased neuronal or network excitability in the early stages of neurodegeneration will be an important driver of pathophysiology. Consequently, there is considerable focus on the potential to target PV+ cells for interventions to modulate excitability and slow, or even halt, disease progression.

## Conclusion

Overall, these intermutually mechanistic insights provide a detailed account of the prevailing role (and vulnerability) of PV+ neurons, particularly highlighting mitochondrial impairment as an emerging pathophysiological mechanism across these major psychiatric and neurodegenerative diseases. The reviewed literature evidence suggests that PV+ neurons may represent an attractive target which could conceivably fuel the vital development of novel, potent therapeutics, with far-reaching applicability.

## Perspectives

- PV+ vulnerability and mitochondrial dysfunction is a shared mechanism across several major psychiatric and neurodegenerative diseases.
- Identification of novel biomarkers specific to PV+ dysfunction and mitochondrial impairment, e.g. miR137-COX6A2, may improve patient stratification in heterogenous diseases, such as in neuropsychiatric disorders, in order to improve clinical trial stratification, monitor disease progression and improve treatment outcome [118].
- Therapeutics which can modify overlapping pathophysiological pathways would be beneficial. PV+ neurons may represent an attractive treatment target with far-reaching applicability.

## Open Access

Open access for this article was enabled by the participation of Newcastle University in an all-inclusive *Read & Publish* agreement with Portland Press and the Biochemical Society under a transformative agreement with JISC.

## Abbreviations

ACC: anterior cingulate cortex
AD: Alzheimer's disease
DLBs: dementia with Lewy bodies
DRE: drug resistant epilepsy
mTOR: mammalian target of rapamycin
NMDA: *N*-methyl-d-aspartate
NMDA-R: NMDA receptor
OXPHOS: oxidative phosphorylation
PCP: phencyclidine
PFC: prefrontal cortex
PMD: primary mitochondrial disease
PTZ: pentylenetetrazole
TLE: temporal lobe epilepsy
